# Synergistic Radiation Protective Effect of Purified *Auricularia auricular-judae* Polysaccharide (AAP IV) with Grape Seed Procyanidins

**DOI:** 10.3390/molecules191220675

**Published:** 2014-12-11

**Authors:** Haina Bai, Zhenyu Wang, Jie Cui, Keli Yun, Hua Zhang, Rui Hai Liu, Ziluan Fan, Cuilin Cheng

**Affiliations:** 1 School of Food Science and Engineering, Harbin Institute of Technology, 73 HuangHe Road, NanGang District, Harbin 150090, China; E-Mails: baihaina@163.com (H.B.); sbleli@163.com (K.Y.); zhhua@hit.edu.cn (H.Z.); ccuilin@hit.edu.cn (C.C.); 2 School of Forestry, Northeast Forestry University, 26 HeXing Road, DongLi District, Harbin 150040, China; E-Mail: fzl_1122@163.com; 3 Department of Food Science, Cornell University, Ithaca, NY 14853, USA; E-Mail: RL23@cornell.edu

**Keywords:** grape seed procyanidins, *Auricularia auricular-judae* polysaccharides, radiation protective, synergistic, splenocytes

## Abstract

The aim of this study was to investigate the synergistic antioxidant potential and protective effect of grape seed procyanidins (GSP) in combination with *Auricularia auricular-judae* polysaccharides (AAP IV) on radiation injury in splenocytes. Rat splenocyte irradiation resulted in significantly higher apoptosis rate, malondialdehyde (MDA) (*p* < 0.005), reactive oxygen species (ROS) (*p* < 0.01); cell viability, total superoxide dismutase (T-SOD) (*p* < 0.01), catalase (CAT) (*p* < 0.01), glutathione peroxidase (GSH-P_X_) (*p* < 0.05), activity and glutathione (GSH) (*p* < 0.01) levels were significantly reduced, compared with the control group. “GSP + AAP IV” treatment of rat splenocytes at doses of “GSP (0.3 μg/mL) + AAP IV (50 μg/mL)” displayed higher radioprotective and antioxidative effects than the administration of either GSP or AAP IV, as evident by lower levels of MDA (*p* < 0.001) concentration, as well as higher cell viability and T-SOD (*p* < 0.05), CAT (*p* < 0.005), GSH-P_X_ (*p* < 0.01) and GSH content compared to the radiation group. In addition, *in vivo* studies have shown that “GSP + AAP IV” significantly ameliorated the decrease of spleen index (*p* < 0.005) and spleen GSH (*p* < 0.005) levels and significantly inhibited the increase of MDA (*p* < 0.005) levels of spleen with radiation-induced damage, compared with the non-treated group. The *in vivo* and *in vitro* results suggested that GSP and AAP IV have a synergistic protective effect against radiation-induced injury by improving the antioxidant and immunomodulation activities.

## 1. Introduction

Radiotherapy is one of the most common treatment modalities for human cancers. In radiotherapy, high doses of ionizing irradiation is used to damage target cells or tissues. However, the irradiation also damages the normal tissues surrounding tumors, therefore, non-target cells and/or tissues should be protected against radiation injury. In particular, the immune system is most sensitive to radiation, as the radiation induces a high level of lymphocyte apoptosis, causes damage to the hematopoietic system, *etc*. [1,2]. Thus radioprotective compounds are of importance in clinical radiation therapy [3]. Though a large variety of compounds have shown promise as radio-protectors in laboratory studies, most of them failed even before reaching the preclinical stage due to their toxicity and side effects. Various natural radioprotective products have been discovered, including polyphenol [4], flavonoids [5], anthocyanins [6] and polysaccharides [7].

Grape seed procyanidins (GSP) is internationally recognized as one of the most effective natural antioxidants for the removal of free radicals in the human body. As a biologically active ingredient, it has a wide range of physiological functions, such as improving blood circulation, protecting eyesight and as an anti-oxidant [8,9], and so on. The simplest procyanidins in GSP are catechin and epicatechin, and other components are the dimers of catechin and epicatechin, and other complex structures like the trimers or tetramers and so on up to decamers. Procyanidins can inhibit the increase of MDA levels induced by radiation [10], improve the immune system [11], and play a protective role against radiation [12,13]. Black fungus is a medicinal edible fungus, that belongs to the basidiomycotina fungi, mainly distributed in the Lesser Khingan Mountains in the northeast region of China. Black fungus polysaccharide not only displays a very high nutritional value, but also has various pharmacological functions. The literature has reported that black fungus polysaccharide has antioxidant, blood lipid lowering [14], anti-tumor and anti-aging [15] activity. In the field of radioprotection, some research reports that the *Auricularia auricular-judae* polysaccharides (AAP) show protective effects against radiation induced injuries of mice [16]. This has aroused more and more attention is its development and utilization.

Targeting multiple pathways by combination of two or more natural products as a new type of radioprotective agent is presumed to better delay the progression of radiation damage [17]. Emerging evidence has shown that a multi-targeting agent or the combination of agents targeting multiple pathways having radioprotective activities without causing any toxicity would be required for the success of prevention and/or treatment of injury induced by γ-irradiation [18,19]. Therefore, we believe that the combination of GSP and AAP, or other agents together would be highly effective in preventing and treating radiation damage. This will be helpful for understanding the effect of the relationship between GSP and AAP, exploring their synergistic effects on cell injury induced by radiation in splenocytes, providing a scientific basis for their application in dietary supplements and drugs for the prevention and treatment of splenocyte damage.

## 2. Results and Discussion

### 2.1. Purification of the AAP

**Figure 1:**
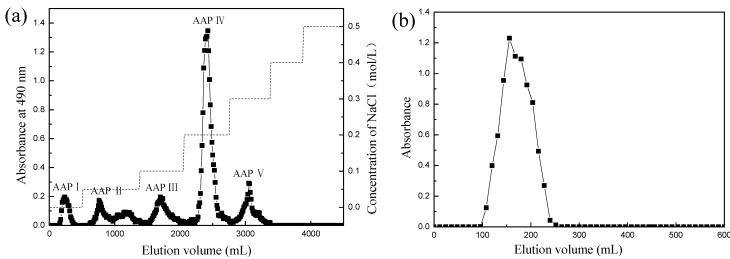
(**a**) The AAP was further separated by DEAE-52 column chromatography into five fractions, namely AAP I, AAP II, AAP III, AAP IV and AAP V; (**b**) The main peak (AAP IV) was further fractionated over Sephadex G-200 column and a component collected, namely AAP IV.

The AAP was further separated by DEAE-52 column chromatography into five fractions by stepwise elution with sodium chloride solutions (0, 0.1, 0.2, 0.3, 0.4 and 0.5 mol/L), namely AAP IV, AAP II, AAP III, AAP IV, and AAP V (Figure 1a). The material recovered from the main peak (AAP IV) was further fractionated over Sephadex G-200 column (Figure 1b), which stressed that AAP IV was a single polysaccharide peak.

### 2.2. Primary Structure Analysis of the AAP IV

**Figure 2:**
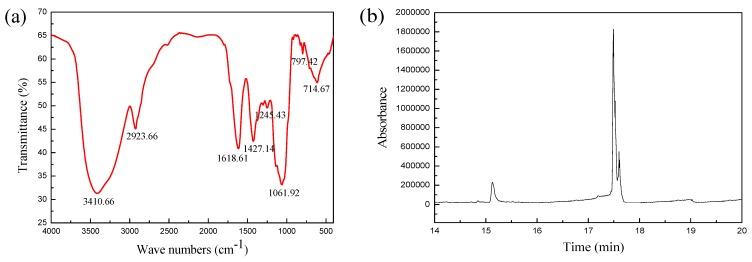
(**a**) FT-IR spectra of AAP IV; (**b**) The GC-MS total ion chromatography of AAP IV.

The molecular weight distribution of AAP IV determined by HPGPC was centered at 3.30 × 10^5^ Da. The GC-MS results showed that AAP IV was composed of d-xylose, d-mannose, d-glucose and d-galactose in a ratio of 1.91:4.67:9.91:0.31 (Figure 2b). The specific optical rotation was ${\left[\alpha \right]}_{D}^{20}$ +24.3°(H_2_O). The FT-IR spectrum obtained from AAP IV (Figure 2a) had peaks at about 3500 cm^−1^ and 500 cm^−1^ in the carbohydrate region. In all spectra (Table 1), the band in the 3410.66 cm^−1^ region corresponds to the hydroxyl stretching vibration of the polysaccharide and the one at 2923.66 cm^−1^ corresponds to a weak C-H stretching vibration [20]. In addition, a characteristic absorption at 1440–1395 cm^−1^ that corresponds to the C-H stretching vibrations of the carboxyl groups of the sugar units was also observed, which signifies that AAP IV was an acidic polysaccharide [15,21]. Figure 2a illustrates the absorptions at 1061.92 cm^−1^ and 1245.4 cm^−1^ which ccorrespond to the C-O-C and C-O-H groups of the pyranose ugar units and verified that the AAP IV contained the pyranose form of glucose. The band in the region of 797.42 cm^−1^ corresponds to the d-glucose pyranose monosaccharide.

**Table 1:** Characteristic FT-IR peaks of AAP IV.

Absorption at Region (cm^−1^)	Vibration Type	Functional Group
3410.66	Stretching vibration of O-H	O-H
2923.66	Stretching vibration of C-H	-CH_2_
1618.61	Variable angle vibration of N-H	-CONH
1427.14	Stretching vibration of C-O	-COOH
1000–1250	Stretching vibration of C-O-C and C-O-H	C-O
797.42	Symmetric stretching vibration of C-O-C	d-glucose pyranose

### 2.3. Cytotoxicity

**Figure 3:**
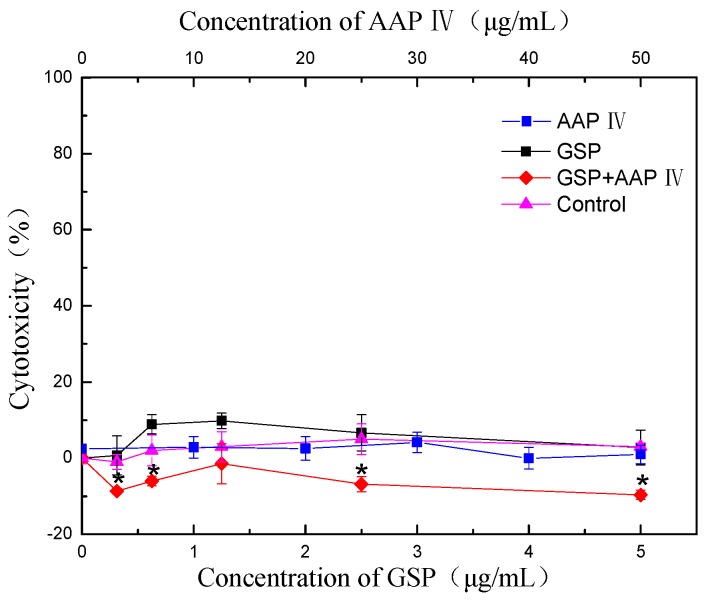
Effect of GSP, AAP, and their combination on cytotoxicity of splenocytes. * indicates a significant difference from the control (*p* < 0.05).

Illustrated in Figure 3 is the cytotoxicity of GSP, AAP IV, and the two-way combination of these two agents (GSP + AAP IV) toward the growth of splenocytes *in vitro*.

No cytotoxicity was observed in GSP at concentrations of ≤5 μg/mL. AAP IV showed no cytotoxicity toward splenocytes at the maximum level of 50 μg/mL. In addition, no cytotoxicity was detected for the combination of GSP (5 μg/mL) plus AAP IV (50 μg/mL).

### 2.4. Splenocyte Viability

**Figure 4:**
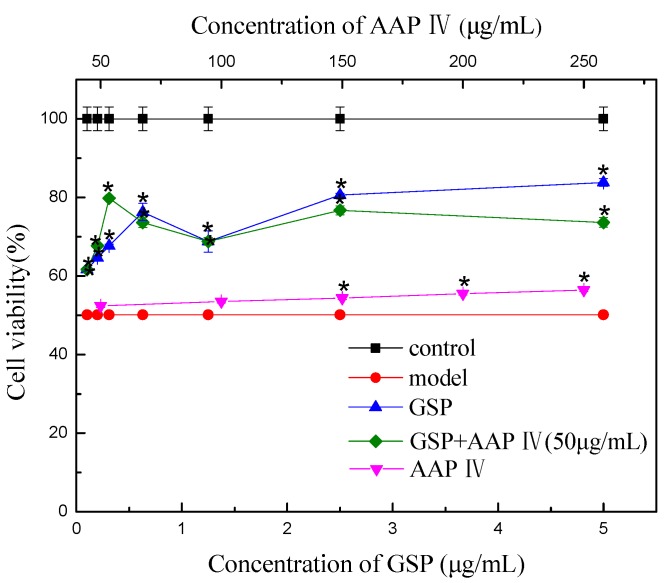
Effect of GSP, AAP IV, and their combination on viability of splenocytes. * indicates a significant difference from the model (*p* < 0.05).

Hematopoietic tissues and the immune system are highly sensitive to radiation. In general, proliferating stem or progenitor cells are particularly vulnerable to radiation-induced cell damage, a prominent example being the stem cells in the hemopoietic systems [22,23]. Several studies have reported that the exposure of animals to radiation leads to the destruction of the lymphoid and hemopoietic systems by increasing apoptosis of not only peripheral blood lymphocytes, but also splenocytes which are known to be radiosensitive cells in the peripheral immune system [24,25,26]. The effect of GSP, AAP IV, and their combination on the viability of splenocytes was examined by an MTT assay. The viability of splenocytes was markedly reduced by an exposure to gamma ray irradiation when compared with control cells (Figure 4). As shown, the viability of splenocytes was increased by the treatment with GSP or “GSP + AAP IV” compared to the radiation (RT) group. Figure 4 and Table 2 show that GSP is mainly responsible for the radiation protection effect.

**Table 2:** Effect and CI of GSP and AAP IV combination on viability of splenocytes.

Combination		Effect	CI
GSP(μg/mL)	AAP IV(μg/mL)		
0.10	50	0.18	1.02
0.20	50	0.35	0.65
0.31	50	0.60	0.11
0.63	50	0.47	0.64
1.25	50	0.37	3.60
2.50	50	0.53	1.43
5.00	50	0.47	5.09

As shown in Table 2, the CI of splenocytes viability between the GSP (0.31 μg/mL) + AAP IV (50 μg/mL) treatment group and their single treatment group was 0.11. The results show that GSP (0.31 μg/mL) and the AAP (50 μg/mL) showed the strongest synergistic effect protecting the splenocytes’ viability. When an extracellular signaling molecule activates a cell surface receptor, signal transduction occurs, and this process involves the actions of numerous cellular messengers. Though different constituents may affect various cellular messengers, the same response may appear in a cell. The mechanisms underlying the synergistic therapeutic actions of herbal medicines are that different agents may regulate either the same or different targets in various pathways, and therefore cooperate in an agonistic or synergistic way [27]. The research found that “GSP + AAP IV” protects cells against their death under irradiation conditions as do other combinations that include polyphenols and polysaccharides [28,29,30,31]. On the other hand, combinations with high concentrations of AAP IV (50 μg/mL) or GSP (1.25 and 5 μg/mL) reduced splenocyte viability, indicating that the combination shows lower radioprotection with a high dose of GSP. Each of the drugs (or compounds) in the total effect of the interaction of the body, is less than the sum of their individual effects, and this state may be due to the fact AAP IV (50 μg/mL) and GSP (1.25 and 5 μg/mL) compete for the same receptor, resulting in a competitive antagonistic effect [32].

### 2.5. Proliferation of Splenocytes

**Figure 5:**
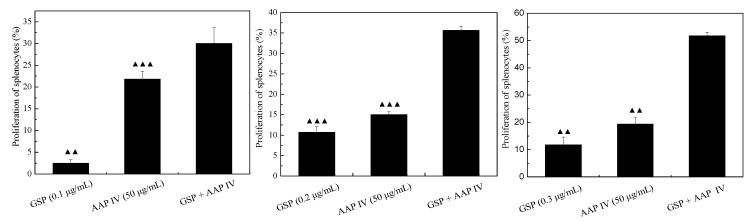
Effect of GSP, AAP IV and their combination on proliferation of splenocytes. ^▲▲^ *p* < 0.01, ^▲▲▲^ *p* < 0.005, as compared with the combination group.

To determine whether GSP, AAP IV, and their combination stimulated splenocyte proliferation an MTT assay was performed. As indicated in Figure 5, the proliferation of splenocytes was markedly increased by treatment with GSP or AAP IV or GSP + AAP IV when compared with the control cells. The GSP + AAP IV combination was greater than the AAP IV with GSP sum of the splenocyte’s proliferation rate. The results show that the role of the GSP and the AAP IV has significant synergistic proliferation of spleen cells.

Polysaccharide biological response modifiers (BRMs) are usually strong mitogens. Macrophages, T-lymphocytes, B-lymphocytes and NK cells proliferate in response to polysaccharide BRM stimulation, which is mediated through the binding of polysaccharides to its corresponding receptors. The receptors and binding proteins of polysaccharide BRMs are derived from the innate immunity system and include TLRs, SRs, β-glucan receptor, CR3, mannose receptor, binding proteins of the lectin binding pathway and the alternative and classical complement pathway [33]. The study of structure–function relationship of polysaccharide BRMs is hampered by the complexity of monosaccharide compositions and glycosidic linkages. Nearly all reported data are restricted to polysaccharide BRMs with simple monosaccharide composition and glycosidic linkages like β-glucan and mannan, and most of the researches was performed on β-glucan.

Recently, some phenolic compounds have been shown to promote specific and non-specific immune responses in different ways [24,34,35]. Previous studies have demonstrated that splenocyte proliferation stimulated by medicinal fruits and vegetables has been attributed to their high concentration of phenolic compounds [36,37]. Our results in this study show the stimulating effect of grape seed procyanidins on splenocyte proliferation. The immunomodulating activity observed in these compounds might be ascribed to the presence of phenolic hydroxy groups or to other molecular moieties [38]. Phenolic compounds have been previously shown to stimulate or suppress the immune system affecting an enzymatic system as the electron-transferring system resulting in an immunomodulating property, especially on phagocytic activity [39]. Some studies show that the procyanidin C1 induces macrophage activation via NF-κB and MAPK pathways, leading to Th1 polarization in murine splenocytes, which suggests that procyanidin C1 regulates innate and adaptive immunity by macrophage activation and Th1 polarization [40].

### 2.6. Apoptosis of Splenocytes

**Figure 6:**
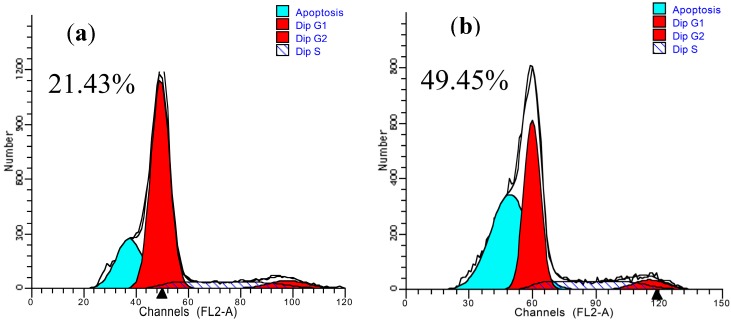

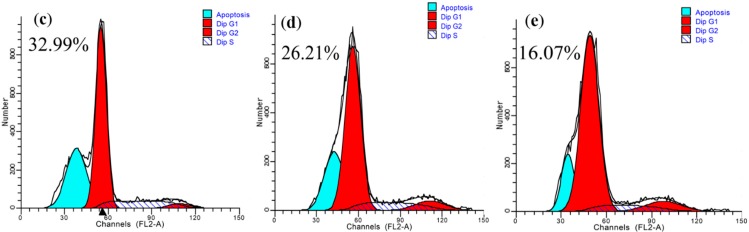
Effect of GSP, AAP IV, and their combination on apoptosis of splenocytes. (**a**) Normal group; (**b**) RT model group; (**c**) GSP (0.3 μg/mL) group; (**d**) AAP IV (50 μg/mL) group; (**e**) GSP (0.3 μg/mL) + AAP IV (50 μg/mL) group.

To identify whether “GSP (0.3 μg/mL) + AAP IV (50 μg/mL)” decreases the proportion of apoptotic sub-G1 hypodiploid cells in 10 Gy-irradiated splenocytes, PI staining assay was used. As indicated in Figure 6, the formation of apoptotic DNA in sub-G1 peak was dramatically increased at 4 h after gamma ray irradiation up to 49.45% ± 0.9% (Figure 6b), compared to non-irradiated cells (21.43% ± 0.1%, Figure 6a), whereas GSP or AAP IV or “GSP + AAP IV” treatment showed an interestingly lower percentage of cells in apoptotic peak at GSP (0.3 μg/mL, Figure 6c), AAP IV (50 μg/mL, Figure 6d) and “GSP (0.3 μg/mL) + AAP IV (50 μg/mL)” (32.99% ± 0.5%, 26.21% ± 0.2% and 16.07% ± 0.2%, respectively, Figure 6e).

These results indicate that “GSP (0.3 μg/mL) + AAP IV (50 μg/mL)” enhanced splenocyte proliferation by synergistically inhibiting the formation of apoptotic DNA caused by gamma ray irradiation. Recent studies have also suggested that phloroglucinol of *Ecklonia cava* protects mice from damages caused by ionizing radiation by proliferation of splenocytes through the inhibition of apoptosis [1].

### 2.7. Product of Splenocytes

Malondialdehyde (MDA), a secondary product of lipid peroxidation, used as an indicator of tissue damage, results from a series of chain reactions. In addition, the resulting radiation hydrolysis from the body’s water formed a large amount of reactive oxygen species (ROS) that attacked different cellular macromolecules such as DNA, lipids, proteins, and induce cell death, including apoptosis [41,42]. γ-Ray exposure caused a significant elevation of ROS and MDA in splenocytes compared with the normal group. The protective effects of GSP and AAP IV against the γ-ray exposure-induced elevation of ROS and MDA are presented in Table 3.

**Table 3:** Effects of GSP, AAP IV, and their combination on splenocytes product level. Values represent the mean and standard deviation of at least three independent replicas. ^▲▲^ *p* < 0.01, ^▲▲▲^ *p* < 0.005, compared with normal control group. ^#^ *p* < 0.05, ^##^ *p* < 0.01, ^###^ *p* < 0.005, ^####^ *p* < 0.001, compared with RT model group. Numbers in parentheses indicate the combination index.

Groups	ROS	MDA (nmol/mg)	GSH (mg/g)
Normal Control	32.49 ± 1.66	7.93 ± 0.30	80.00 ± 2.12
RT model group	47.07 ± 1.59 ^▲▲^	17.84 ± 0.45 ^▲▲▲^	57.03 ± 3.76 ^▲▲^
RT + GSP (0.1 μg/mL)	46.16 ± 1.68	16.19 ± 0.90	61.61 ± 3.83
RT + GSP (0.2 μg/mL)	45.77 ± 1.70	14.45 ± 0.78 ^#^	61.30 ± 4.01
RT + GSP (0.3 μg/mL)	44.38 ± 1.60	12.50 ± 0.55 ^##^	64.19 ± 5.55
RT + AAP IV (50 μg/mL)	42.18 ± 1.33	15.97 ± 0.65 ^#^	60.65 ± 6.25
RT + AAP IV (100 μg/mL)	40.65 ± 1.84 ^#^	14.31 ± 0.58 ^#^	61.53 ± 2.35 ^##^
RT + GSP (0.1 μg/mL) + AAP IV (50 μg/mL)	41.92 ± 1.58 ^##^ (1.01)	13.56 ± 0.50 ^##^ (0.81)	68.36 ± 5.34 ^###^ (0.23)
RT + GSP (0.2 μg/mL) + AAP IV (50 μg/mL)	39.16 ± 1.66 ^###^(0.38)	10.51 ± 0.56 ^###^(0.50)	63.64 ± 5.04 ^###^ (0.94)
RT + PGS (0.3 μg/mL) + AAP IV (50 μg/mL)	38.78 ± 1.78 ^###^(0.38)	8.95 ± 0.45 ^####^ (0.32)	73.80 ± 0.13 ^###^ (0.13)

The administration of GSP did not cause significant changes in the splenocytes, as the ROS and MDA content decreased in the RT-treated splenocytes. However, the administration of a combination of GSP and AAP IV significantly prevented the elevation of splenocyte ROS and MDA content induced by RT treatment in a dose-dependent manner. At a dose of 0.3 μg/mL GSP in combination with 50 μg/mL of AAP IV, splenocyte ROS and the MDA content decreased 17% and 50% compared with RT model control, and the CIs of ROS and MDA content between their combination and the single treatment were all less than 1. Recent studies have demonstrated that the cellular damages caused by ROS generation might cause adecrease of cell viability and the improvement of cell proliferation by phloroglucinol and eckol is related with their inhibitory action on ROS production [1,24].

GSH is an extremely efficient intracellular buffer for oxidative stress and GSH acts as a non-enzymatic antioxidant that reduces H_2_O_2_, hydroperoxides (ROOH) and xenobiotic toxicity [43]. The GSH content (*p* < 0.01) decreased significantly in the model group when compared with the normal control group. The GSP or AAP IV did not affect the GSH content in the RT-treated splenocytes. However, the combination of GSP with the AAP IV at a dose of GSP (0.3 μg/mL) + AAP IV (50 μg/mL), significantly (*p* < 0.005) increased the GSH concentration when compared with the model group. The CI of GSH concentration between the “GSP (0.3 μg/mL) + AAP IV (50 μg/mL)” treatment group and their single treatment group was 0.13 (see Table 2). Data indicate that treatment with thymol or curcumin prior to radiation exposure increased GSH levels and decreased the level of MDA [44,45]. In addition, the significant increase in GSH protects cellular proteins against oxidative damage through the glutathione redox cycle and also directly detoxifies ROS induced by irradiation.

### 2.8. Enzyme Levels of Splenocytes

A major defense mechanism involves the antioxidant enzymes, including T-SOD, CAT, and GSH-Px which convert active oxygen molecules into low or harmless substances [15]. These enzymes prevent generation of hydroxyl radicals and protect the cellular constituents from oxidative damage. A reduction in the activities of these enzymes is associated with the accumulation of highly reactive free radicals, leading to deleterious effects such as loss of integrity and function of cell membranes.

**Figure 7:**
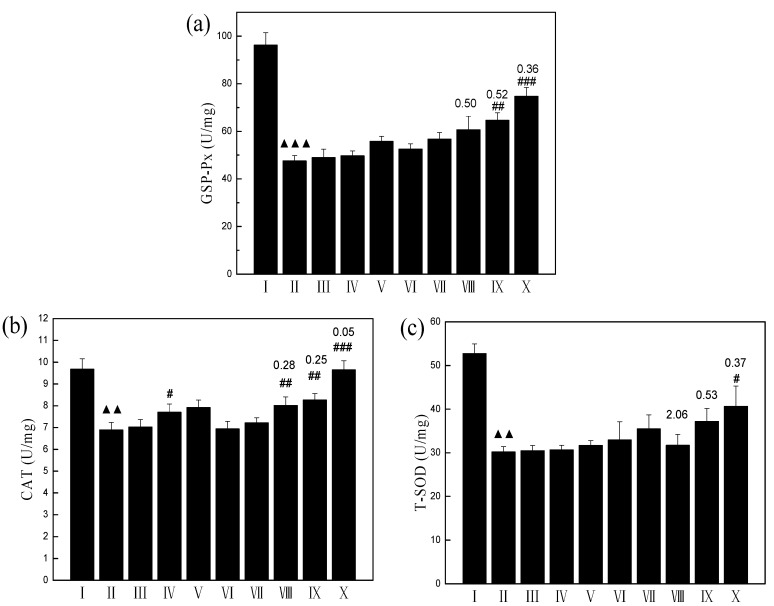
(**a**) Effect of GSP, AAP IV, and their combination on GSH-Px activity of splenocytes; (**b**) Effect of GSP, AAP IV, and their combination on CAT activity of splenocytes; (**c**) Effect of GSP, AAP IV, and their combination on T-SOD activity of splenocytes. ^▲▲^ *p* < 0.01, ^▲▲▲^ *p* < 0.005 compared with normal control group; ^#^ *p* < 0.05, ^##^ *p* < 0.01, ^###^ *p* < 0.005, compared with RT model group; Group I: Normal Control; Group II: RT model group; Group III:RT + GSP (0.1 μg/mL); Group IV: RT + GSP (0.2 μg/mL); Group V: RT + GSP (0.3 μg/mL); Group VI: RT + AAP IV (50 μg/mL); Group VII: RT + AAP IV (100 μg/mL); Group VIII: RT + GSP (0.1 μg/mL) + AAP IV (50 μg/mL); Group IX: RT + GSP (0.2 μg/mL) + AAP IV (50 μg/mL); Group X: RT+ GSP (0.3 μg/mL) + AAP IV (50 μg/mL); numbers on the cylindrical indicate the combination index; Values represent the mean and standard deviation of at least three independent replicas.

As shown in Figure 7a, the glutathione peroxidase (GSH-Px) activity in the RT model group was significantly reduced by 50% when compared with the normal control group. The GSP or the AAP IV did not affect GSH-P_X_ activity in RT model splenocytes. However, administration of the GSP (0.3 μg/mL) + AAP IV (50 μg/mL) significantly (*p* < 0.05) increased the GSH-P_X_ activity from 47.58 (U/mg) to 74.67 (U/mg). The CI value of GSH-P_X_ activity between the GSP (0.3 μg/mL) + AAP IV (50 μg/mL) treatment group and their single treatment group was 0.36.

The catalase (CAT) activity (*p* < 0.01) significantly decreased in the RT model group when compared with the control group. GSP or AAP did not affect GSH content in splenocytes exposed to radiation treatment. However, the combination of GSP with AAP IV at dose of GSP (0.3 μg/mL) + AAP IV (50 μg/mL) (*p* < 0.005) significantly increased the CAT concentration when compared with the RT model group. The CI value of CAT activity between the GSP (0.3 μg/mL) + AAP IV (50 μg/mL) treatment group and their single treatment group was 0.05 (see Figure 7b).

As shown in Figure 7c, the splenocyte total superoxide dismutase (T-SOD) activity in the RT model group was significantly reduced by 42% when compared with the normal control group. GSP or AAP IV did not affect the T-SOD activity in the RT model splenocytes. However, administration of GSP (0.3 μg/mL) + AAP IV (50 μg/mL) (*p* < 0.05) significantly increased the T-SOD activity from 30.44 (U/mg) to 40.64 (U/mg). The CI value of T-SOD activity between GSP (0.3 μg/mL) +AAP IV (50 μg/mL) treatment group and their single treatment group was 0.37.

Radiation treatment resulted in decreased T-SOD, CAT and GSH-Px activity, GSP, AAP IV and their combination treatment caused T-SOD, CAT and GSH-Px increases, especially in the group treated with 0.3 μg/mL GSP combined with 50 μg/mL AAP IV. It has been suggested that ROS are responsible for radiation-induced toxicity, therefore destruction of ROS by SOD and CAT would ameliorate such toxicity, which means that the enzymes would be able to scavenge the generated ROS [46,47].

### 2.9. Spleen Indices

**Figure 8:**
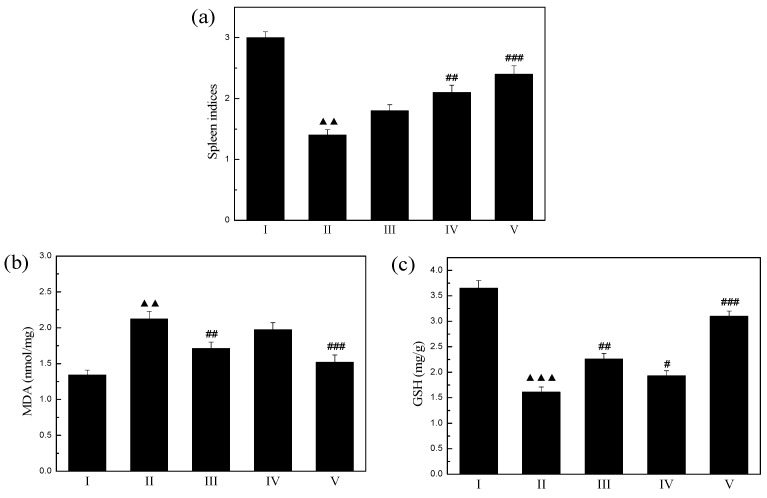
(**a**) Effect of GSP, AAP IV, and their combination on spleen indices; (**b**) Effect of GSP, AAP IV, and their combination on spleen MDA level; (**c**) Effect of GSP, AAP IV, and their combination on spleen GSH level. Values represent the mean and standard deviation of at least three independent replicas. ^▲▲^ *p* < 0.01, ^▲▲▲^ *p* < 0.005, compared with normal group. ^#^ *p* < 0.05, ^##^ *p* < 0.01, ^###^ *p* < 0.005, compared with RT model group. Group I: Normal group; Group II: RT model group; Group III: RT + GSP (1.2 mg/kg BW/D); Group IV: RT + AAP IV (200 mg/kg BW/D); Group V: RT + GSP (1.2 mg/kg BW/D) + AAP IV (200 mg/kg BW/D).

The spleen is the largest immune organ in the body. Its weight could reflect well the bodily damage induced by irradiation. Radioprotectors could increase the weight of this organ [48]. As shown in Figure 8a, the spleen index of the model group mice (1.40 ± 0.09 mg/g) (*p* < 0.01) was much lower than that of normal group mice (3.01 ± 0.10 mg/g). After the treatment with GSP, AAP IV, and their combination, the spleen indexes were all increased. The spleen index of GSP + AAP IV combination (2.40 ± 0.14 mg/g) was higher than that of AAP IV (2.13 ± 0.12 mg/g) or GSP (1.8 ± 0.10 mg/g). The results show that the GSP and AAP IV synergistically and significantly increased the spleen index of treated mice.

### 2.10. Spleen Products

MDA is an index of lipid peroxidation and oxidative stress. As shown in Figure 8b, radiation markedly increased the content of MDA from 1.34 ± 0.07 to 2.21 ± 0.11 nmol/mg, and this effect was inhibited significantly by GSP (1.71 ± 0.09 nmol/mg) (*p* < 0.05) and AAP IV (1.91 ± 0.10 nmol/mg) (*p* < 0.01). The content of MDA was significantly decreased in the combination group (1.52 ± 0.10 nmol/mg) compared model group.

As shown in Figure 8c, the radiation group (1.61 ± 0.10 mg/g) showed significantly (*p* < 0.01) decreased GSH levels compared with the control group (3.65 ± 0.15 mg/g). When the mice were treated with a combination of GSP and AAP IV (3.10 ± 0.10 mg/g), the content of GSH had a stronger increase than with GSP (2.26 ± 0.11 mg/g) or AAP IV (1.93 ± 0.10 mg/g) separately (*p* < 0.005).

## 3. Experimental Section

### 3.1. Chemicals and Reagents

The GSP, at 95% plus purity, was purchased from Zhengzhou Lion Biotechnology Co., Ltd., Zhengzhou, China. The DEAE-52 and Sephadex-G200 were both purchased from Amersham Pharmacia Company, Shanghai, China. The 3-(4,5-dimethylthiazol-2-yl)-2,5-diphenyltetrazolium bromide (MTT) and dimethyl sulfoxide (DMSO) were purchased from Sigma Chemical Co., St. Louis, MO, USA. The Roswell Park Memorial Institute 1640 (RPMI-1640) was from Hyelone Chemical Co., Logan, UT, USA. The catalase (CAT), superoxide dismutase (SOD), glutathione peroxidase (GSH-P_X_), reduced glutathione (GSH), malondialdehyde (MDA) and protein quantization measurement kits were purchased form Nanjing Jiancheng Bioengineering Institute, Nanjing, China. The reactive oxygen species (ROS) measurement kits were purchased from the Beyotime Institute of Biotechnology, Jiangsu, China.

### 3.2. Preparation and Purification of the AAP

The black fungus were purchased from the Antarctic market, Harbin, China. The black fungus were weighed and then immersed in 60 volumes of clean distilled water at room temperature for 24 h, followed by an ultrasound-assisted extraction with hot (100 °C) water for 5 h. The residue was extracted twice using the same procedure. The resulting suspension was then centrifuged (4000 r/min for 10 min) and concentrated on a rotary evaporator under reduced pressure at 50 °C. The concentrated supernatants were then precipitated with three volumes of absolute ethanol (95%) and maintained overnight at 4 °C. The resulting precipitate was then separated by centrifugation, washed exhaustively with 95% alcohol, dissolved in deionized water, and then lyophilized to produce a crude polysaccharide extract [49]. This preparation, for the purpose of this research, was called “crude polysaccharide”. The dried powder was dissolved in 80 °C water bath with distilled water, then protein was removed by the Sevage method, by adding 1/5 volumes of Sevage reagent (chloroform-butyl alcohol = 5:1), at centrifuging at 4000 r/min for 15 min. The denatured protein was removed by repeating the procedure at least four times until all the denatured protein was completely removed. The supernatant, with the protein removed, was concentrated under by adding three times the volume of 95% ethanol and then precipitating the mixture for 24 h, using a vacuum drying oven, vacuum drying at 40 °C to obtain the polysaccharide extract (designated as AAP).

Further fractionation was performed using anion exchange chromatography. The AAP was dissolved in a H_2_O and then membrane-filtered (0.45 μm). The solution was then applied to a column (2.6 cm × 60 cm) of DEAE-52 pre-equilibrated with H_2_O. Fractions were obtained by stepwise elution with increased ionic strength of NaCl (0, 0.1, 0.2, 0.3, 0.4, and 0.5 mol/L) at a flow rate of 0.2 mL/min. The main fraction was eluted with 0.3 mol/L NaCl solution, as quantified by the phenol-sulfuric acid method. The major fractions obtained were pooled, concentrated, and then lyophilized to give the *Auricularia auricula-judae* polysaccharide coded as AAP IV. It was then collected and further purified by gel filtration chromatography on a Sephadex G-200 column (2.6 cm × 60 cm). The AAP IV was then dissolved in the minimal volume of H_2_O and added to the column; and eluted with H_2_O at a flowrate of 0.2 mL/min, collected by automatic collection in 12.5 mL tubes, which were quantified by the phenol-sulfuric acid method.

### 3.3. Infrared Spectroscopy Analysis

An infrared spectrum of AAP IV was recorded using a Fourier transform infrared spectrophotometer (PerkinElmer, Waltham, MA, USA). The sample was ground with spectroscopic grade potassium bromide (KBr) powder and then pressed into 1 mm pellets for FT-IR determination in the frequency range of 4000–400 cm^−1^.

### 3.4. Analysis of Monosaccharide Composition by GC-MS

#### 3.4.1. Hydrolysis

Hydrolysis was carried out with TFA. The purified polysaccharide sample (15 mg) was hydrolyzed with 4 mL of 2 M TFA at 110 °C for 4 h in a sealed glass tube. After hydrolysis, the solution was evaporated to dryness at 50 °C, and then a stream of nitrogen and methanol (3 mL) were used to remove the excess acid. This procedure was repeated 5 times to remove the TFA completely. After that, the hydrolyzed products were ready for the following derivatization.

#### 3.4.2. Derivatization

A reducing reaction must be carried out before derivatization, through which the aldoses in the standard solution or in the hydrolyzed sample are reduced to their corresponding alditols. The reducing reaction was performed at room temperature for 3 h by adding sodium borohydride (25 mg). Several drops of glacial acetic acid were then added to stop the reaction until air bubbles disappeared and then the solution was evaporated to dryness by rotary evaporator at 50 °C. Methanol (4 mL) and a stream of nitrogen gas were used to remove the reducing agent five times, and then the residue was dried at 110 °C for 30 min to remove the moisture. The acetylation was carried out with acetic anhydride (3 mL) and pyridine (1 mL) in a water bath at 100 °C for 5 h. After that, the mixture was evaporated to dryness at 80 °C and trichloromethane (5 mL) was added to dissolve the residue. The organic phase was washed with distilled water (5 mL) for 5 times to remove the impurities. Finally, the water was removed with anhydrous sodium sulfate and the organic phase was transferred into a GC vial for GC-MS analysis.

#### 3.4.3. GC-MS Analysis

GC-MS was used for separation of monosaccharides. A capillary column DB-5 (30 m × 0.25 mm I.D., 0.25 μm film thickness) was used, with helium as carrier gas at a constant flow of 1.0 mL/min. The temperature program was the following: initial temperature 80 °C, and held for 2 min, 10 °C/min ramp to 200 °C, and held for 2 min. The total analysis time was 20 min and the equilibration time 2 min. The temperature of the injection port was 240 °C and a 1 μL volume was injected in splitless mode. The mass spectrometer was operated in electron ionization mode with an ionizing energy of 70 eV, ion source temperature 150 °C, MS Quad temperature 230 °C, electron multiplier voltage (EM Volts) 1750 V when performing selected ion monitoring, scanning from *m/z* 35 to 450.

### 3.5. Preparation of Splenocytes

Wistar rats were anaesthetized using diethyl ether and euthanized by cervical dislocation, then immersed in 75% ethanol for 3 to 5 min, and placed on sterile plates; operating on a clean sterile bench, the spleen was isolated and aseptically removed by mincing with a sterile forceps and scissors and then homogenized with a syringe needle core through a 76 μm mesh screen to obtain a homogeneous cell suspension. The splenocytes were harvested by centrifugation (1000  r/min, for 10 min), and the red blood cells were lysed with a lysis buffer. The cells were washed twice with phosphate-buffered saline (PBS), then suspended in a complete RPMI 1640 medium with 10% fetal calf serum and 100 U/mL antibiotics, adjusted to 5 × 10^6^ cells/mL. The cell incubation was conducted at 37 °C, in 5% CO_2_ enriched atmosphere. Cell viability was determined by 0.4% Trypan blue dye exclusion, and purified cells (viability ˃ 90%) were used directly for the experiments [50].

### 3.6. Irradiation with ^60^Coγ-Rays

The radiation center of the Heilongjiang Academy of Agricultural Sciences was used for the irradiation experiments. The splenocytes received ^60^Coγ-radiation (10 Gy) at a dose rate of 1.59 Gy/min and disposable radiation time 8.36 min at a source-to-cell plates distance (midpoint) of 130 cm. Following irradiation, the cells were maintained in 10% serum containing media in a 5% CO_2_ atmosphere at 37 °C.

### 3.7. Splenocytes Viability Assay

The viability of splenocytes was examined by a colorimetric MTT assay, which depends on the conversion of yellow tetrazolium bromide to its purple formazan derivative by mitochondrial succinate dehydrogenase in viable cells [51]. Cells were treated with GSP and AAP IV for 12 h and then exposed to γ-rays at 10 Gy. Then, 24 h later, 10 μL of the MTT stock solution (5 mg/mL) was added to each well. After incubation for 4 h, the plate was centrifuged (1000 *g*, 10 min) followed by the aspiration of the supernatants. Formazan crystals present in each well were dissolved in 100 μL of DMSO, and the absorbance was determined at 570 nm. The cell viability (%) = value for RT experimental well/(value for normal group control well − value for RT model well) × 100.

### 3.8. Proliferation of Splenocytes

The proliferation of splenocytes was examined by an MTT assay. Concanavalin A (5 mg/mL) was added to appropriate wells. The cells were treated with samples and 72 h later, 10 μL of the MTT stock solution (5 mg/mL) was added to each well. After incubation for 4 h, the plate was centrifuged (1000 *g*, 10 min), followed by a aspiration of the supernatants. Formazan crystals, present in each well, were dissolved in 100 μL of DMSO, the absorbance was determined at 490 nm. The cell proliferation (%) = (value for experimental well − value for normal group control well)/value for normal group control well × 100.

### 3.9. PI (Propidium Iodide) Staining Assay

To identify whether samples decreased the proportion of apoptotic sub-G1 hypodiploid cells in 10 Gy-irradiated splenocytes, a PI staining assay was performed. Splenocytes (1 × 10^7^ cells/well) were treated with samples and 12 h later exposed to γ-rays at 10 Gy. Then, 4 h later, the cells were collected and washed in cold PBS. After centrifugation, the cells were suspended in solution containing 100 μg/mL of PI and 100 μg/mL of ribonuclease A (RNase A) for 30 min. The cells were analyzed by assessing the proportion of Sub-G1 contents using a BD FACSCalibur TM flow cytometer (BD Biosciences, San Jose, CA, USA).

### 3.10. 2'-7'-Dichlorofluorescein Diacetate (DCF-DA) Assay

To detect the production levels of intracellular reactive oxygen species (ROS), a DCF-DA assay was performed. Splenocytes (1 × 10^7^ cells/well) were treated with samples for 2 h and then exposed to γ-rays at 10 Gy. Then, 4 h later, the cells were collected and washed in cold PBS. After incubation, 10 μM of DCF-DA solution was added to each well for 20 min at 37 °C, the cells were then collected and washed in cold PBS. After centrifugation, the cells were then suspended in PBS. The intensity of 2,7-dichlorofluorescein was measured at 585 and 620 nm using a BD FACSCalibur TM flow cytometer.

### 3.11. Animals

Male KM mice (6–8 weeks old, weighing 18–22 g each) were obtained from the Laboratory Animal Center of Harbin Medical University, Harbin, China and were housed under specific pathogen-free conditions. The animal room was controlled for temperature (22 ± 2 °C), light (12 h light/dark cycles) and humidity (50% ± 10%). Rodent laboratory chow pellets and tap water were randomly supplied. The experimental protocol was approved by Institutional Animal Ethical committee.

### 3.12. Experimental Design and Oral Administration

This experiment was conducted as follows:

Group I: Normal mice (Normal group)
Group II: Radiation (RT Model group)
Group III: Radiation + GSP (1.2 mg/kg BW/D)
Group IV: Radiation + AAP IV (200 mg/kg BW/D)
Group V: Radiation + GSP (1.2 mg/kg BW/D) + AAP IV (200 mg/kg BW/D)

Fifty healthy male mice were randomly divided into seven groups. Group I and Group II served as normal control and radiation control. The samples were administered using vehicle solution (deionized water). The animals in the normal and RT model groups serving as normal control and radiation control only received deionized water orally. After two weeks of treatment, the animals received the full body radiation of ^60^Co (6 Gy). Then the animals were fasted overnight prior to being sacrificed by decapitation. The spleen was removed promptly and weighed, and stored at −80 °C for further analysis.

### 3.13. Analysis of Spleen Indices

To analyze the spleen index, the spleen of the mice was surgically removed and weighed. The spleen indice was calculated in accordance with the following formula according to the method described previously [52]: Spleen index (mg/g) = weight of spleen (mg)/body weight (g).

### 3.14. Determination of Biological Parameters of Splenocytes and Spleen

The splenocytes were collected and washed with PBS three times, frozen after thawing, and this process is repeated three times. A cell or spleen homogenate was used for the estimate of CAT, SOD activities, and GSH, MDA, GSH-P_X_, ROS and protein content; all were estimated using kits according to the manufacturers’ instructions.

### 3.15. Combination Index for Determining Addition, Synergism, and Antagonism

The CI based on the classic isobologram equation [53] has been used for data analysis of two-way combination as:

(1) $$\text{CI}=\frac{{\left(\text{D}\right)}_{1}}{{\left({\text{D}}_{\text{x}}\right)}_{1}}+\frac{{\left(\text{D}\right)}_{2}}{{\left({\text{D}}_{\text{x}}\right)}_{2}}$$

where (D)_1_ and (D)_2_ are the doses of GSP and AAP IV, respectively, in the combination system; (D_x_)_1_ and (D_x_)_2_ are the doses of GSP and AAP IV alone, respectively. For data analysis of combinations, CI < 1, CI = 1, and CI > 1 indicate synergistic, additive, or antagonistic effects, respectively [54,55]. The combination index is calculated by drug concentration and effect using the Calcusyn software.

The calculation method of effective numerical is as follows:

(2) $$F\text{a}=\frac{\text{ΔΑ}}{\text{ΔΒ}}$$

where *Fa* was the fraction affected by the dose; ΔA was the difference between the drug-treated group and the radiation model group; ΔB was the difference between the control group and the radiation model group.

### 3.16. Statistical Analysis

All statistical analyses employed SPSS for Windows, Version 13.0. Data were expressed as means ± standard deviation. Statistical analyses were performed by one-way ANOVA. Differences at *p* < 0.05 were considered statistically significant by Duncan’s New Multiple-Range Test.

## 4. Conclusions

The present study investigated the antioxidant, immunomodulatory and radioprotective activities of GSP, AAP IV, GSP + AAP IV*.* The data from the CI results revealed that GSP, when combined with AAP IV, could significantly increase the effects of GSH-Px, CAT, T-SOD and GSH, and could lower the ROS and MDA levels in splenocytes exposed to ^60^Co-radiation when compared to the irradiated, non-treated controls. Furthermore, “GSP + AAP IV” could improve the immunomodulatory activities.
